# Supplementary material for: Comparing the effects of dynamic and holding isometric contractions on cardiovascular, perceptual, and near-infrared spectroscopy parameters: A pilot study
Source: PLoS One. 2023 Feb 16;18(2):e0281885. doi: 10.1371/journal.pone.0281885 (PMC9934453; doi:10.1371/journal.pone.0281885)
Supplement: Attachment — Submitted filename: ResponseToReviewers.docx [file pone.0281885.s001.docx]

Dear Editor,

Thank you for the opportunity to improve the manuscript, a special thanks to Reviewers for all comments, suggestions, and corrections. We have modified the manuscript accordingly, and highlighted (blue) the changed parts. Also, a detailed point-by-point correction is listed below.

Reviewer #1: General:

The writing is generally excellent, and the study is interesting and unique.

Of course, the most obvious issue with the paper is the sample size of 7. Even if this is a ‘pilot’, I often suggest to authors to simply ‘keep going’ and collect more data. The introduction and methods section would not have to change, and depending on the ‘new’ results, the discussion may only change slightly. Since this is not a training/longitudinal study, I see little reason why the study cannot/should not be continued until a more suitable sample size is achieved. If for whatever reason, this is not possible, then the authors should clearly state why collection was stopped after only 7 participants.

- The sample size was increased to 10 participants.

Title:

The title could be clearer. At present, readers may be unsure if you are testing different kinds of isometric contraction (holding vs pushing, for example. I suggest the title be changes to something like:

“A comparison of dynamic and holding isometric contractions on cardiovascular, perceptual and near-infrared spectroscopy parameters: A pilot study”

- The title was improved and is clearer now. “Comparing the effects of dynamic and holding isometric contractions on cardiovascular, perceptual, and near-infrared spectroscopy parameters: A pilot study”. Please see lines 1-4.

Abstract:

The abstract is well written, and makes me want to read more.

The first thing that comes to mind is that it seems odd that sig differences were only seen for select measure and sets, instead of after each set. This makes me think that finding were potentially random chance, which is of course an issue with small samples/’pilot’ studies. I hope the authors address these questions in the body of the article.

- We have edited the text accordingly. The concept of holding was added to the text, as well as some minor changes in the sample description data and statistical results. Please see the Abstract.

Introduction:

Line 32: remove the period before reference 1.

- We have edited the text accordingly. Please see line 33.

Lines 32-33: I do not believe that the semitendinosus is a prime mover, but is more of a synergist. Feel free to argue the point, but perhaps move the semitendinosus with the other muscles as a synergist, or stabilizer etc.

- We have edited the text accordingly. During this exercise, the *vastus lateralis* (VL) muscle act as primary mover, *longissimus* (LG) and *semitendinosus* (ST) muscles act as stabilizers, and *soleus* (SL) muscle act as secondary capacity. Please see lines 33-35.

Paragraph 2 is excellent. However, it would be valuable to briefly mention the difference between holding and pushing isometrics, and how holding are particularly understudies, despite arguably being more practical/easy to do in a typical weightroom setting.

- We have edited the text accordingly and we had to add new references related to the characterisation of the different modes of isometric contractions. In fact, we completely agree that the information clarifying what type of isometric contraction is being studied is extremely relevant. Please see lines 40-45.

Paragraph 3 is to technical, and brings up too many points that are not particularly relevant to the study/paper as a whole (i.e., melanin). I suggest simplifying this paragraph, and sticking to the points that are critical, such as deoxy, and re-oxy, and why those might be things practitioners may care about.

- We have edited the text accordingly. Some relevant information related to NIRS has been moved to the methodology chapter. Please see lines 59-61 and 81-82.

I also suggest making the purpose statement, and hypothesis its own small paragraph.

- We have edited the text accordingly. Please see lines 66-71.

Methods:

The methods seem good, though to be honest, MOXY and other tools like that are far from my expertise. I hope the other reviewer(s) is knowledgeable in this regard.

Good work using the Bonferroni post hoc. I often see underpowered studies skipping the correction at all, or using more lenient corrections such as Tukey’s etc.

It should be clear why/when means vs medians were used for reporting. Did this have to do with distribution/the Shapiro-Wilk results?

- We have edited the text accordingly. We could not agree more that it should be explained why averages and medians appear, since the averages are when the data has a normal distribution and the medians are when the data does not have a normal distribution. Please see lines 182-186 and 190-193.

Results:

Remove the spaces between numbers and the ‘%’ sign. I.e., ‘47.7 %’ should be ’47.7%’.

- We have edited the text accordingly. Please see lines 204, 205, 208, 209, 210, 214, 215.

Some interesting results, esp the perceived exertion and HR going opposite directions between the two conditions. Since the SL is not a prime mover, perhaps relating back to some of the literature comparing pushing vs holding isometrics could be valuable to the reader. Ie., scientist such as Schaefer and Bittmann, and Roger Enoka’s group have generally found more activation and/or hemodynamic response in the supporting/synergist muscles during holding contractions, whereas the prime mover is more active/affected in the pushing isometrics.

- We have edited the text accordingly. Some differences between holding and pushing were emphasized. The amplitude of variation of the mechanical muscular oscillations seems to be greater during holding muscle action in relation to pushing muscle action in muscles that present stabilizing function and in prime movers it is the inverse. Please see lines 262-267.

Discussion:

Generally I wish the authors tried to better explain why some of the response differences were only seen in one set.

- We have edited the text accordingly. According to the available literature, the physiological rational is related to hyperaemia in response to exercise. Please see lines 270-272.

First and second paragraphs can be combined.

- We have edited the text accordingly. Please see line 244-254.

The small paragraph on lines 252-255 can be combined with the paragraph above it. The authors make good points about needing EMG etc. to further uncover the findings.

- Since additional information has been included, we did not consider it necessary to add it to the paragraph above. We await feedback.

Generally try to avoid paragraphs that are 2 sentences or less, and try to incorporate them into other paragraphs.

- We have edited the text accordingly.

While I understand that is difficult to understand fully, it would be interesting for the authors to try and explain why the ISO resulted in higher RPE, whereas the dynamic resulted in higher HR; esp since the 6-20 RPE scale was created to corelate with heartrate (60-200 BPM). Anecdotally/in my experience, participants tend to feel almost ‘board’, or even believe they are not doing anything useful during ISO contractions, and therefore may feel less motivated. Where it is clear they are ‘accomplishing’ something during DYN contractions. This may play into perceptions.

- We have edited the text accordingly. Please see lines 312-317 and 321-326.

The most important limitation is missing. SAMPLE SIZE. Please be VERY clear about this, even so, I highly recommend continuing this study until the sample is into the double digits.

- The sample size was increased to 10 participants.

Figures/Tables:

Nice, no need for change or additional figures or tables.

**Additional information from the authors:**

Abstract

- The results, in descriptive and numerical terms, were rectified for all variables.

Material and methods | Participants

- The results, in numerical terms, were rectified for all variables.
- One information that was in the introduction was changed for this topic. Please see lines 81-82.

Results

- The results, in descriptive and numerical terms, were rectified for all variables.

Discussion

- The results, in descriptive terms, were rectified.

References

- New references have been added, duly marked.

Figures

- Figure 3 was elaborated again, changing only the results.
